# Evaluation of the Effectiveness of Regulatory Measures for the Use of Natural Ecological Space Based on the Remote Sensing‐Driven PLUS‐InVEST Model: A Case Study From the Central Mountainous Area of Hainan Island

**DOI:** 10.1002/ece3.72025

**Published:** 2025-10-16

**Authors:** Jiceng Xu, Xiaodong Mu, Ziwei Ma

**Affiliations:** ^1^ Hainan Research Academy of Environmental Sciences Haikou China

**Keywords:** central mountainous area of Hainan Island, habitat quality, InVEST model, PLUS model, tropical rainforest area

## Abstract

Unreasonable reclamation and construction activities have led to a decline in habitat quality and loss of biodiversity in tropical rainforest areas, especially in key biodiversity regions globally. To evaluate the effectiveness of natural ecological space use regulation measures, in this study, the remote sensing data‐driven PLUS‐InVEST coupled model was used to evaluate and forecast the habitat quality and degree of degradation in the central mountainous region of Hainan Island, a global biodiversity hotspot. By setting natural ecological space use control measures, namely Ecological Conservation Redline, the spatiotemporal variations in land use/land cover and habitat quality were investigated under two regulatory scenarios: natural development (ND) and ecological priority (EP). The study revealed that: (1) Under the natural development (ND) regulatory scenario, from 2007 to 2021, artificial ecosystems such as farmland, water bodies, and construction land showed an increasing trend, while natural ecosystems such as forests, shrubs, and grasslands exhibited a decreasing trend in the study area. (2) Between 2007 and 2021, the habitat quality in the study area exhibited a decreasing trend, with a total reduction of 3.12%. (3) From 2021 to 2035, compared to the natural development (ND) scenario, under the ecological priority (EP) regulatory scenario, influenced by the Ecological Conservation Redline, the area of natural ecosystems in the study area showed an increasing trend, with an overall improvement in habitat quality by 0.34%, an increase in excellent quality habitat area of 57.12 km^2^, and a significant improvement in habitat quality levels. The research results confirmed the effectiveness of implementing strict natural ecological space use control measures in enhancing ecosystem protection and habitat quality in the central mountainous region of Hainan Island, providing theoretical support for ecosystem conservation and habitat quality improvement in island tropical rainforest areas.

## Graphical abstract

Unreasonable reclamation and construction activities have led to a decline in habitat quality and loss of biodiversity in tropical rainforest areas, especially in key biodiversity regions globally. In this study, the remote sensing data‐driven PLUS‐InVEST coupled model was used to evaluate and forecast the habitat quality and degree of degradation in the central mountainous region of Hainan Island, a global biodiversity hotspot. By setting natural ecological space use control measures, namely ecological protection redlines, the spatiotemporal variations in land use/cover and habitat quality were investigated under two regulatory scenarios: natural development (ND) and ecology priority (EP). The research results confirmed the effectiveness of implementing strict natural ecological space use control measures in enhancing ecosystem protection and habitat quality in the central mountainous region of Hainan Island, providing theoretical support for ecosystem conservation and habitat quality improvement in island tropical rainforest areas.

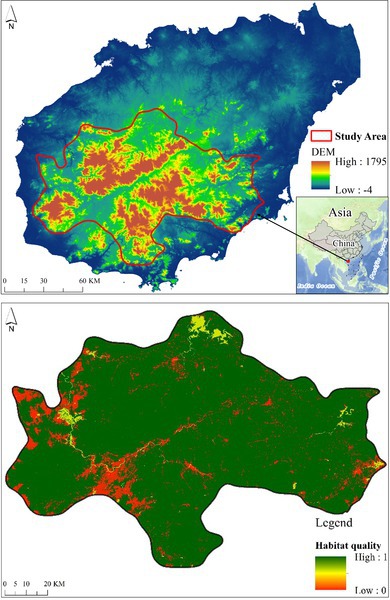

## 1 Introduction

Tropical rainforests are one of the most biologically rich ecosystems on Earth (Alvarado‐Robledo et al. 2024; Zhu 2023). They play a crucial role in maintaining the ecological balance and climate stability of Earth (Z. Fu et al. 2018). However, with the intensifying human activities, the habitat quality of tropical rainforests is facing severe challenges (Bourgoin et al. 2024; Dallaqua et al. 2021). Therefore, a deep analysis of the spatio‐temporal changes in habitat quality of tropical rainforests is of great significance for the protection of this rare ecological resource.

Both Convention on Biological Diversity (CBD 2014; UNDP 2015) and Transforming Our World: The 2030 Agenda for Sustainable Development (UNDP 2015) advocate for the assessment of ecosystem conservation status (Hall 2019; Jørgensen 2015), especially the scientific assessment of habitat quality (HQ), which has significant impacts on biodiversity conservation, human well‐being, and the stability of natural ecosystems. Habitat quality (HQ) refers to the ability of an ecosystem to provide sustainable survival and reproduction for species. High‐level habitat quality (HQ) and a healthy ecological environment are crucial for the long‐term and high‐quality development of the socio‐economy. However, development and construction, as well as farmland reclamation, have adverse effects on biodiversity (Jung et al. 2019). The rapid expansion of development, construction, and farmland reclamation has encroached upon the habitats of various species, resulting in a decline in habitat quality and biodiversity (Deng et al. 2023; Mondal, Bandyopadhyay, et al. 2024; Tang et al. 2020). Habitat quality is an important indicator reflecting the level of regional biodiversity (L. Zheng et al. 2023). Currently, numerous habitat suitability models have been researched and developed internationally, with the most common being the HIS (Shan et al. 2022), C‐Plan (Winchester and White 2022), and Maxent (Xing et al. 2023) habitat suitability models. Nevertheless, these models require species biodiversity data and information on the number of species present in the environment, making their application challenging. The Integrated Valuation of Ecosystem Services and Trade‐offs (InVEST) model is widely used due to its advantages such as easy access to data, simple operation, and straightforward data processing (Dong et al. 2022; Wei et al. 2022). Furthermore, the InVEST model driven by remote sensing data has proven to be advantageous in land use/land cover and ecological environment management (S. Li et al. 2024; Mondal, Naskar, et al. 2024). The remote sensing data‐driven model has been successfully used to assess habitat quality (Wang and Cheng 2022), the impact of urban and farmland expansion on habitat quality (Tang et al. 2020), and to predict future habitat quality through simulation (Lei et al. 2022).

One of the significant consequences of human activities is the evolution of land use/land cover, and the frequency of human activities also shapes the pattern of land use/land cover change (B. Chen et al. 2022). Land use/land cover is a crucial factor in habitat quality (HQ) assessment, and its variation not only affects the improvement and degradation of HQ but also further impacts biodiversity levels (Li, Ma, and Zhou 2022; Potapov et al. 2024). Therefore, considering land use/land cover transitions in studying the dynamic evolution of habitat quality may help maintain biodiversity. In the field of land use/land cover change research, current models for land use/land cover simulation and prediction based on remote sensing data drive mainly include the Cellular Automata (CA) model (Rahnama 2021) and the CA‐Markov model (F. Fu et al. 2022), as well as models further developed based on the CA model, such as the Logistic‐CA model (Y. Chen et al. 2014), Artificial Neural Network (ANN)‐CA model (Roy et al. 2024; C. Zhang et al. 2021), Future Land Use Simulation (FLUS) model (Liu et al. 2017), and Patch‐generating Land Use Simulation (PLUS) model (Liang et al. 2021). Among the aforementioned models, the PLUS model stands out with the highest simulation accuracy and has been widely applied in land use/land cover research by scholars (L. Gao et al. 2022; Wang, Li, et al. 2022). For instance, the remote sensing data‐driven PLUS model was employed to investigate the driving factors of sustainable land expansion in Wuhan, China (Liang et al. 2021); the PLUS model, driven by remote sensing data, was used to predict landscape ecological risks in the Fujian Delta region, effectively evaluating land use efficiency (S. Zhang et al. 2022); and by integrating the remote sensing data‐driven PLUS model with Guangzhou's “14th Five‐Year” transportation plan, the land use pattern of Guangzhou in 2030 based on natural development and transportation planning scenarios was simulated (S. Lin and Wang 2023).

As mentioned above, remote sensing data have been widely applied to drive the InVEST model for habitat quality (HQ) assessment and the PLUS model for land use/land cover simulation. Yet, research on utilizing remote sensing technology to drive the coupled PLUS‐InVEST model for evaluating natural ecological space use regulation measures is still relatively scarce. Consequently, the effectiveness of natural ecological space use regulation measures is assessed using the remote sensing data‐driven PLUS‐InVEST model.

Being a significant ecological functional zone in China and recognized as one of the global biodiversity hotspots (Hou et al. 2018; Lei et al. 2023), the central mountainous region of Hainan Island stands as a relatively isolated island‐type geographical area, housing the largest tropical rainforest in China. In the past few decades, the expedited development of Hainan as an International Tourism Island and a Free Trade Port, along with intensified human activities like construction and agriculture, has led to increased habitat fragmentation (Lei et al. 2022). Therefore, exploring the spatio‐temporal changes in historical and future land use/land cover and habitat quality in the central mountainous area of Hainan Island is a pivotal case study for researching island‐type mountainous tropical rainforest areas.

In response to such challenges, the Chinese government proposed the national strategy of “Ecological Conservation Redline” nationwide in 2011, aiming to establish an ecological protection framework and safeguard national and regional ecological security (J. Gao et al. 2020). The “Implementation Plan for the National Ecological Civilization Pilot Zone (Hainan)” was released in 2019, with the implementation of differentiated land use regulation becoming a central task in the development of the National Ecological Civilization Pilot Zone (Hainan). Via the “Three Zones and Three Lines” delineation work in the “Hainan Provincial Territorial Spatial Planning (2021–2035),” the boundaries of the Ecological Conservation Redline were determined, and the central mountainous region of Hainan Island was clearly defined as an ecological protection area, with strict protection of its natural ecosystems. Especially in 2021, the Hainan Rainforest National Park was officially established, providing a higher level of institutional safeguard for the ecological protection of the central mountainous region of Hainan Island. Although the “Hainan Provincial Territorial Spatial Planning (2021–2035)” has been approved by the State Council of the People's Republic of China, and the Hainan Rainforest National Park was established in 2021. To date, there has been no comprehensive exploration of the impact of historical and future land use/land cover changes on habitat quality in the central mountainous region of Hainan Island. Consequently, it is imperative to investigate the effects of land use/land cover changes on habitat quality in the central mountainous regions of Hainan Island.

This study employs remote sensing data to drive the integrated PLUS‐InVEST model, to analyze the spatiotemporal changes in land use/land cover and habitat quality in the central mountainous region of Hainan Island, and to predict the spatial patterns of future land use/land cover and habitat quality under two regulatory scenarios. The specific research objectives are: (1) To analyze the spatiotemporal pattern of land use/land cover in the central mountainous region of Hainan Island from 2007 to 2021 under a natural development (ND) regulatory scenario and to assess the spatiotemporal pattern of habitat quality; (2) To simulate the spatial distribution pattern of land use/land cover and habitat quality in the central mountainous region of Hainan Island in 2035 under scenarios of natural development (ND) and ecological priority (EP); (3) To evaluate the effectiveness of regulatory measures for the use of natural ecological space in the central mountainous region of Hainan Island.

## 2 Materials and Methods

### 2.1 Overview of Study Area

**FIGURE 1:**
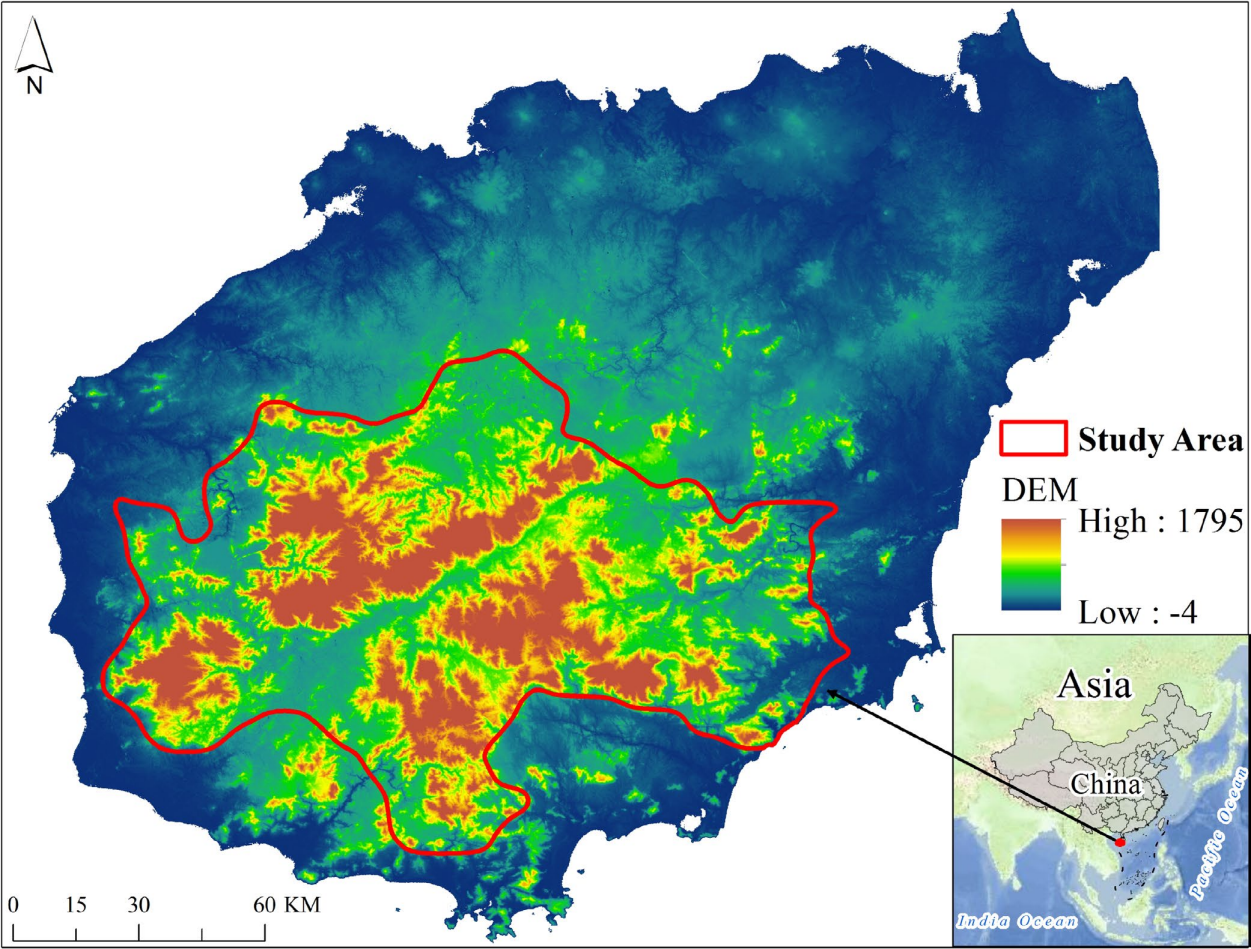
Map of the Central Mountainous Area of Hainan Island.

The central mountainous area of Hainan Island is situated in the central part of Hainan Province (the main island) in China, spanning from 108°44′ to 110°20′ E in longitude and from 18°21′ to 19°25′ N in latitude. The area is characterized by mountainous and hilly terrain. Dominated by medium–low mountains (elevation range approximately 0 ~ 1867 m, with Wuzhi Mountain peak at 1867 m), this area features an average slope of 15.6°, forming a concentric zonal landform of “central mountain core–low mountains/hills–platform plains.” The region falls within the monsoon climate zone at the northern fringe of tropical Asia, featuring a tropical mountainous climate with ample rainfall and a humid environment. The annual mean temperature ranges from approximately 23.1°C to 24.8°C, with an annual average precipitation of roughly 1288 to 2417 mm. The area is home to the largest tropical rainforest in China, exhibiting a typical tropical rainforest ecosystem (Zhu and Zhou 2002). Additionally, the Hainan Tropical Rainforest National Park has been established here (Li, Tang, et al. 2022), protects approximately 149 nationally key plant species and 145 key animal species. Vegetation exhibits complete altitudinal zonation: tropical lowland rainforest (< 800 m), montane rainforest (700 ~ 1300 m), tropical coniferous forest (> 1200 m), and alpine cloud forest (> 1300 m). Dominated by latosols, the soil vertical zones progress from latosol to lateritic red earth, yellow earth, and meadow soil with elevation. With a total area of approximately 11,213 km^2^, the central mountainous area of Hainan Island occupies roughly one‐third of the island's total surface, forms headwaters for major rivers (Nandu, Changhua, Wanquan). This region is considered a key ecological functional area in China (H. Zheng et al. 2019) and is recognized as one of the critical regions for global biodiversity (Han et al. 2024). This area plays a crucial role in maintaining the ecological balance of Hainan Island, protecting endangered species (e.g., Hainan gibbons), mitigating natural disasters, and safeguarding the island's ecosystem security. The study area is illustrated in Figure 1.

### 2.2 Methodology

#### 2.2.1 Land Use/Land Cover (LULC) Transition Matrix

The land use/land cover transition matrix is employed to depict the alterations in land use/land cover types throughout the research duration. The land use/land cover type transition matrix signifies the transformations in various land use/land cover categories across a designated timeframe. The formula for the land use/land cover type transition matrix is given below:

$$S_{\mathrm{ij}}=\left[\begin{array}{cccc} S_{11} & S_{12} & \cdots & S_{1n}\\ S_{21} & S_{22} & \cdots & S_{2n}\\ \vdots & \vdots & \vdots & \vdots \\ S_{n1} & S_{n2} & \cdots & S_{\mathrm{nn}} \end{array}\right]$$

where $S_{\mathrm{ij}}$ is the area; n is the number of land use/land cover types; i,j are various land use/land cover types at the beginning and end of the study area, respectively.

#### 2.2.2 PLUS Model

The PLUS model (patch‐generating land use simulation model) was developed by Liang Xun et al. at China University of Geosciences (Liang et al. 2021). The PLUS model integrates the Land Expansion Analysis Strategy (LEAS) for rule mining and a Cellular Automata (CA) model based on a multiple random seeds mechanism. Utilizing land use/land cover data from 2007 and 2021 in the study region, the expansion patterns of seven land use/land cover types were ascertained. Additionally, the development probabilities for each type were derived using the random forest algorithm. Considering the principle of data availability, 16 driving factors for land use/land cover types were selected, including population density, GDP, distance to transportation facilities, annual average temperature, annual average precipitation, DEM, slope, aspect, soil type, distance to water systems, etc. (Table 1). The resolution and projection of all 16 driving factors align with the land use/land cover data of the study area.

**TABLE 1:** Data sources for study area, land use/land cover and driving factors.

No.	Category	Data	Year^a^	Resolution	Data resource
1.	The boundaries of the study area	Biodiversity protection and water conservation functional area in central Hainan	2015	—	“National Ecological Function Zoning (Revised Edition)” (issued by the Ministry of Environmental Protection and the Chinese Academy of Sciences in November 2015)
2.	Land use/land cover data	Land use/land cover types in the central mountainous region of Hainan Island	20072021	30 m	(Yang and Huang 2022). The 30 m annual land cover datasets and its dynamics in China from 1990 to 2021 [dataset]. In Earth System Science Data (1.0.1, Vol. 13, Number 1, pp. 3907–3925). Zenodo. https://doi.org/10.5281/zenodo.5816591
3.	Restricted conversion zones	Ecological Conservation Redline of Hainan Province	2022	—	Department of Natural Resources and Planning of Hainan Province
4.	Socioeconomic driver	Population density	2020	1 km	WorldPop (www.worldpop.org—School of Geography and Environmental Science, University of Southampton; Department of Geography and Geosciences, University of Louisville; Departement de Geographie, Universite de Namur) and Center for International Earth Science Information Network (CIESIN), Columbia University (2018). Global High Resolution Population Denominators Project—Funded by The Bill and Melinda Gates Foundation (OPP1134076). https://doi.org/10.5258/SOTON/WP00674
5.	GDP	2020	500 m	zhang degang, cheng bo, Chen jinfen, et al. A dataset of spatial distribution of GDP in Hainan island at 500 m from 2012 to 2020[DS/OL]. V1. Science Data Bank, 2022[2023‐08‐16]. https://cstr.cn/31253.11.sciencedb.j00001.00402. CSTR:31253.11.sciencedb.j00001.00402.
6.	Distance to highway	2021	30 m	OpenStreetMap (https://www.openstreetmap.org/)
7.	Distance to trunk road			
8.	Distance to primary road			
9.	Distance to secondary road			
10.	Distance to tertiary road			
11.	Distance to railway			
12.	Distance to railway station			
13.	Climatic and environmental driver	Annual average temperature		2007–2020 (15‐year average)	China Meteorological Data Service Centre (http://data.cma.cn)
14.		Annual average precipitation			
15.		DEM		2016	NASA SRTM1 v3.0
16.		Slope			
17.		Aspect			
18.		Soil type	2009	30 m	Institute of Soil Science, Chinese Academy of Sciences
19.		Distance to water bodies	2020	1:2000	Water bodies in the land use sourced from the Department of Natural Resources and Planning of Hainan Province

^a^ It is permissible to collect driving factors from various time periods (Liang et al. 2021), but ensure that the time period of the driving factors is as close as possible to the time period of the land use/land cover data.

#### 2.2.3 Scenario Settings

The demand for land use/land cover types in the 2035 natural development (ND) scenario is forecasted using the Markov chain module within the PLUS model, based on trends observed in land use/land cover changes between 2007 and 2021. In the 2035 ecologically prioritized (EP) scenario, the primary goal is to limit transformations of forest, shrubland, grassland, and water body types within the Ecological Conservation Redline. This is based on historical development trends and aligned with the requirements outlined in the “Ecological Conservation Redline Environmental Supervision Measures (Trial)” (2022) and the “Hainan Provincial Territorial Spatial Planning (2021–2035).” These guidelines stress the preservation of the Ecological Conservation Redline by ensuring “no reduction in area, no change in nature, and no degradation of function”, along with strict supervision and management. Using the Markov quantitative prediction model and based on the transition probabilities of various land use/land cover types from 2007 to 2021, the transition probabilities of farmland to forest, shrubland, grassland, and water areas are increased by 50%. The transition probabilities of forests to other land use/land cover types, except water areas, are reduced by 60%. The transition probabilities of shrublands to land use/land cover types other than water and forest are decreased by 40%. The transition probabilities of grasslands to other land use/land cover types, apart from water, shrubland, and forest, are lowered by 40%. The transition probabilities of water areas to land use/land cover types other than shrubland, forest, and grassland are reduced by 40%. The PLUS model is used in combination with parameters such as field weights and conversion restrictions to predict the areas of various land use/land cover types under the ecologically prioritized (EP) scenario.

#### 2.2.4 InVEST Model

The InVEST model is developed by Sharp et al. from Stanford University (Wang and Cheng 2022). The habitat quality model is utilized to evaluate the effects of human activities on the ecological environment. Based on previous research (Lei et al. 2022), the parameters for the InVEST model were determined (Table 2 and Table 3). Utilizing these parameters, the habitat quality and the extent of habitat degradation in the central mountainous area of Hainan Island were computed for the years 2007, 2021, and 2035. The calculation of habitat quality and the degree of habitat degradation is based on the following equations.

**TABLE 2:** Habitat threat factors and their attributes.

Threat factors	Max affect distance/km	Weight	Spatial decay type
Farmland	1	0.4	Linear
Construction land	10	1	Exponential

**TABLE 3:** The sensitivity of different land use/land cover types to habitat threat factors.

Code	LULC	Habitat quality	Sensitivity	
			Farmland	Construction Land
1	Farmland	0.3	0	0.6
2	Forests	1	0.6	0.5
3	Shrubs	0.9	0.7	0.8
4	Grasslands	0.7	0.8	0.6
5	Water bodies	0.7	0.2	0.3
6	Construction Land	0	0	0
7	Barren	0.2	0.1	0.9

Formula for Habitat Quality:

$$Q_{\mathrm{xj}}=H_{\mathrm{xj}}\times \left[1-\left(\frac{D_{\mathrm{xj}}^{2}}{D_{\mathrm{xj}}^{2}+k^{2}}\right)\right]$$

where $Q_{\mathrm{xj}}$ is the habitat quality of grid x in land use/land cover type j; $D_{\mathrm{xj}}$ is the degree of habitat degradation, representing the degree of habitat degradation of grid x in land use/land cover type j; $H_{\mathrm{xj}}$ is the habitat suitability of grid x in land use/land cover type j; k is the half‐saturation constant. The value of $Q_{\mathrm{xj}}$ ranges between 0 and 1, a $Q_{\mathrm{xj}}$ value close to 0 indicates low habitat quality, and a $Q_{\mathrm{xj}}$ value close to 1 indicates high habitat quality.

Formula for habitat degradation degree:

$$D_{\mathrm{xj}}=\sum_{1}^{r}\sum_{1}^{y}\left(\frac{\omega_{r}}{\sum_{r=1}^{n}\omega_{r}}\right)\times r_{y}\times i_{\mathrm{rxy}}\times \beta_{x}\times S_{\mathrm{jr}}$$

where $\omega_{r}$ is the weight of each threat factor; $r_{y}$ is the intensity of the threat factor; $i_{\mathrm{rxy}}$ is the influence intensity of threat factor r at grid y of habitat x; $\beta_{x}$ is the anti‐interference level of the habitat; $S_{\mathrm{jr}}$ is the relative sensitivity of different habitats to different threat factors. The degree of habitat degradation ranges from 0 to 1, and a $D_{\mathrm{xj}}$ value close to 1 indicates a higher degree of habitat degradation.

The calculation formula for the influence intensity $i_{\mathrm{rxy}}$ of threat factor r at grid y of habitat x is as follows:

$$i_{\mathrm{rxy}}=1-\left(\frac{d_{\mathrm{xy}}}{d_{\max}}\right)\;\text{if}\;\mathrm{lin}\mathrm{ear}$$

$$i_{\mathrm{rxy}}=\exp\left(-\left(\frac{2.99}{d_{\max}}\right)\times d_{\mathrm{xy}}\right)\;\text{if exponential}$$

where $d_{\mathrm{xy}}$ is the linear distance between grids x and y; $d_{\max}$ is the maximum effective distance of threat factor r.

The natural breaks method in ArcGIS was employed (He et al. 2023) to categorize the habitat quality index value $Q_{\mathrm{xj}}$ into four levels, specifically, excellent, good, medium, and low, as shown in Table 4.

**TABLE 4:** Classification of habitat quality assessment results.

Level	Excellent	Good	Medium	Low
Habitat quality	1> $Q_{\mathrm{xj}}$≥ 0.984	0.984>$\;Q_{\mathrm{xj}}$≥ 0.698	0.698> $Q_{\mathrm{xj}}$≥ 0.298	0.298> $Q_{\mathrm{xj}}$≥ 0
Description	The habitat quality is excellent	The habitat quality is good	The habitat quality is at a medium level	The habitat quality is relatively low

### 2.3 Data Sources and Data Processing

The primary data sources utilized in this research comprise the study area scope, land use/land cover data, data on restricted conversion zones (specifically, the Ecological Conservation Redline in Hainan Province), socio‐economic data, as well as climate and environmental data. The boundaries of the study area were derived from the “National Ecological Function Zoning (Revised Edition)” (issued by the Ministry of Environmental Protection and the Chinese Academy of Sciences in November 2015), specifically the biodiversity protection and water conservation functional zone located in central Hainan. The land use/land cover data were sourced from Zenodo (https://zenodo.org/) and cover two time periods: 2007 and 2021. The data on restricted conversion zones were obtained from the Ecological Conservation Redline of Hainan Province, provided by the Department of Natural Resources and Planning of Hainan Province. The socio‐economic dataset comprises population density data from Worldpop (www.worldpop.org), GDP figures from the Science Data Bank, and transportation information (including railways, highways, etc.) sourced from OpenStreetMap (https://www.openstreetmap.org/). The climate and environmental dataset encompasses annual average temperature, annual average precipitation, DEM, slope, aspect, soil type, and the distance to water bodies. Temperature and precipitation data were sourced from the China Meteorological Data Service Centre, and the annual average temperature and annual average precipitation were calculated using spatial interpolation techniques. Elevation data came from the National Aeronautics and Space Administration (https://www.nasa.gov), while slope and aspect data were derived from the elevation dataset. The soil type information was sourced from the Institute of Soil Science, Chinese Academy of Sciences, while the water system data were provided by the Department of Natural Resources and Planning of Hainan Province. Distances to roads and water bodies were computed using the Euclidean distance tool in ArcGIS software. The entire dataset was cropped based on the vector boundary, projection, and resolution specific to the study area. For detailed data sources, refer to Table 1.

## 3 Results

### 3.1 Analysis of LULC Changes From 2007 to 2021

From 2007 to 2021, significant changes occurred in the land use/land cover types in the central mountainous area of Hainan Island. The total area of forests, shrubs, grasslands, and barren has shown a decreasing trend, while the total area of farmland, water areas, and construction land has shown an increasing trend.

In 2007 and 2021, the land use/land cover type structure in the study area was dominated by forests, accounting for 92.98% and 89.27% of the total area, respectively. The dominant forests showed a decreasing trend, with a reduced area of 415.95 km^2^ and a reduction ratio of 3.99%. The grassland area decreased from 12.47 km^2^ to 1.76 km^2^, showing a decreasing trend with a reduced area of 10.70 km^2^ and a reduction rate of 85.88%. The shrub area decreased from 4.14 km^2^ to 2.47 km^2^, showing a decreasing trend with a reduced area of 1.68 km^2^ and a reduction rate of 40.52%.

**FIGURE 2:**
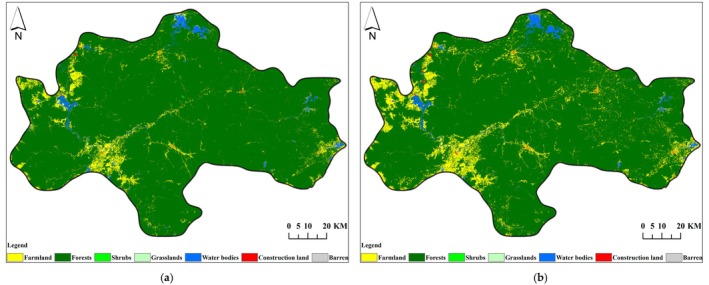
Distribution map of LULC (land use/land cover) in the Central Mountainous Area of Hainan Island in 2007 (a) and 2021 (b).

In 2007 and 2021, the farmland in the study area was 607.64 km^2^ and 1017.08 km^2^, respectively, accounting for 5.42% and 9.07% of the total area, ranking second and showing an upward trend with an increase of 67.38%. The water area was 151.10 km^2^ and 161.78 km^2^, accounting for 1.35% and 1.44% of the total area, respectively, showing an increasing trend with an increased area of 10.68 km^2^ and an increase rate of 7.07%. The construction land showed an increasing trend, with an increase in area from 11.18 km^2^ to 19.46 km^2^ and an increase rate of 74.08%. See Table 5 and Figure 2.

**TABLE 5:** Area and proportion of LULC (land use/land cover) in the Central Mountainous Area of Hainan Island from 2007 to 2021.

Code	LULC	2007		2021		Changes in 2007–2021	
		Area/km^2^	Proportion	Area/km^2^	Proportion	Area/km^2^	Proportion
1	Farmland	607.64	5.42%	1017.08	9.07%	409.45	67.38%
2	Forests	10,426.25	92.98%	10,010.30	89.27%	−415.95	−3.99%
3	Shrubs	4.14	0.04%	2.47	0.02%	−1.68	−40.52%
4	Grasslands	12.47	0.11%	1.76	0.02%	−10.70	−85.88%
5	Water bodies	151.10	1.35%	161.78	1.44%	10.68	7.07%
6	Construction land	11.18	0.10%	19.46	0.17%	8.28	74.08%
7	Barren	0.14	0.00%	0.06	0.00%	−0.07	−53.90%

From 2007 to 2021, the conversion between different land use/land cover types in the central mountainous area of Hainan Island was significant, with a converted area of 652.53 km^2^, accounting for 5.82% of the total area, mainly concentrated between farmland and forests.

**FIGURE 3:**
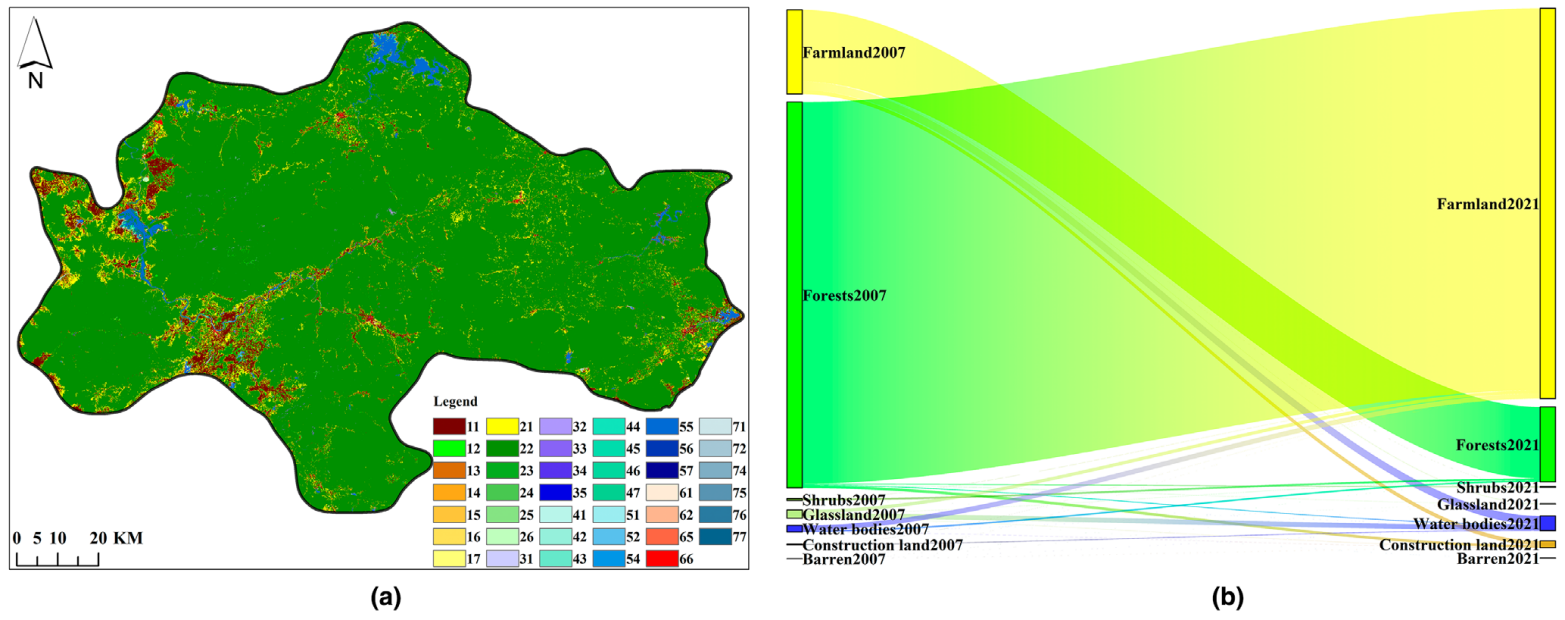
Trajectory (a) and Sankey diagram (b) of LULC (land use/land cover) transfers in the central mountainous area of Hainan Island from 2007 to 2021. Note of trajectory (a):1–7 represent farmland, forest, shrubland, grassland, water area, construction land, and others, respectively. Code 12 indicates that farmland has been converted to forest, and other codes follow the same rule.

The largest transfer area was from forest to farmland (coded as 21), with an area of 510.74 km^2^, accounting for 4.55% of the total area. Among this, 81.16% of the area was distributed across five counties/cities, namely Ledong County, Dongfang City, Qiongzhong County, Wanning City, and Baisha County, with areas of 136.52 km^2^, 111.72 km^2^, 72.71 km^2^, 48.70 km^2^, and 44.88 km^2^, accounting for 26.73%, 21.87%, 14.24%, 9.54%, and 8.79%, respectively. The second largest transfer area was from farmland to forests (coded as 12), with an area of 95.69 km^2^, accounting for 0.85% of the total area. Among this, 79.09% of the area was distributed across five counties/cities: Ledong County, Wanning City, Baisha County, Dongfang City, and Changjiang County, with areas of 29.89 km^2^, 13.07 km^2^, 11.46 km^2^, 10.80 km^2^, and 10.46 km^2^, accounting for 31.23%, 13.66%, 11.98%, 11.28%, and 10.93%, respectively. The third largest transfer area was from farmland to water area (coded as 15), with an area of 11.16 km^2^, accounting for 0.10% of the total area, mainly distributed across three counties/cities: Danzhou City, Dongfang City, and Wanning City. See Figure 3.

### 3.2 Habitat Quality and Degree of Habitat Degradation

**FIGURE 4:**
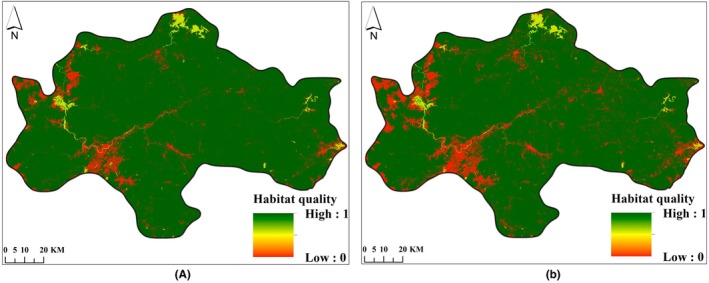
Habitat quality distribution map of the Central Mountainous Area of Hainan Island in 2007 (a) and 2021 (b).

**FIGURE 5:**
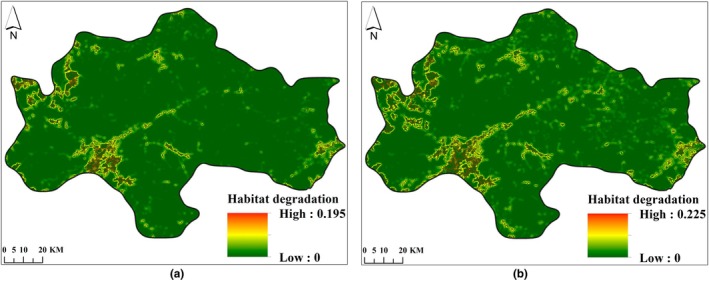
Habitat degradation degree distribution map of the Central Mountainous Area of Hainan Island in 2007 (a) and 2021 (b).

Habitat quality and habitat degradation in the study area in 2007 and 2021 were determined using the InVEST model. See Figures 4, 5.

#### 3.2.1 Dynamic Changes in Habitat Quality and Degree of Habitat Degradation

From 2007 to 2021, the excellent and good habitat quality areas in the central mountainous region of Hainan Island were mainly distributed in higher altitude areas of counties and cities such as Qiongzhong County, Baisha County, Ledong County, and Wuzhishan City. This area was a concentrated distribution area of forests and water areas. The areas of medium habitat quality were concentrated in the farmland areas of Ledong County and Dongfang City, where the habitat suitability and habitat quality were higher than those of construction land areas. Areas with low habitat quality were concentrated in construction land areas. Based on field verification results, there was a correlation between habitat quality (HQ) and major land use/land cover types, with HQ decreasing in the order: forest>water area>farmland > construction land (Table 6). The average habitat quality of the study area in 2007 and 2021 was 0.9563 and 0.9298, respectively. The habitat quality decreased by 3.12% during the period from 2007 to 2021. See Figure 4.

**TABLE 6:** Habitat quality (HQ) validation results for central mountainous areas of Hainan Island based on field survey data.

2021	Field data in 2020			
HQ derived from remote sensing‐driven InVEST model	LULC (land use/land cover)	Point longitude	Point latitude	On‐site aerial images
1.00	Forest	109.372871	18.982848	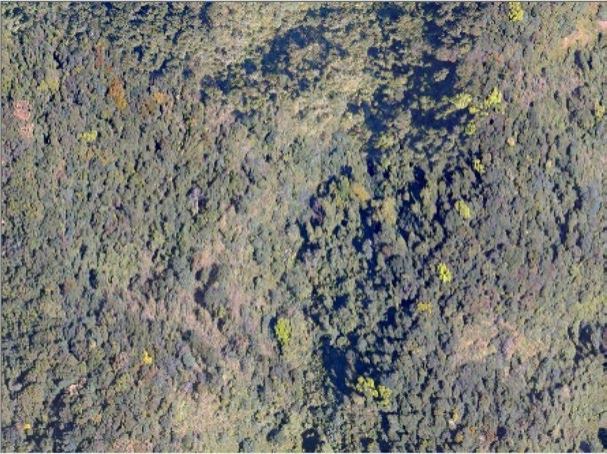
0.70	Water area	109.03996	18.980658	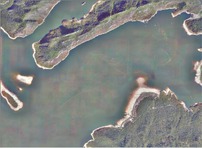
0.30	Farmland	109.202448	18.68981	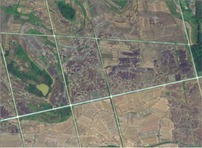
0.00	Construction land	109.836284	19.036786	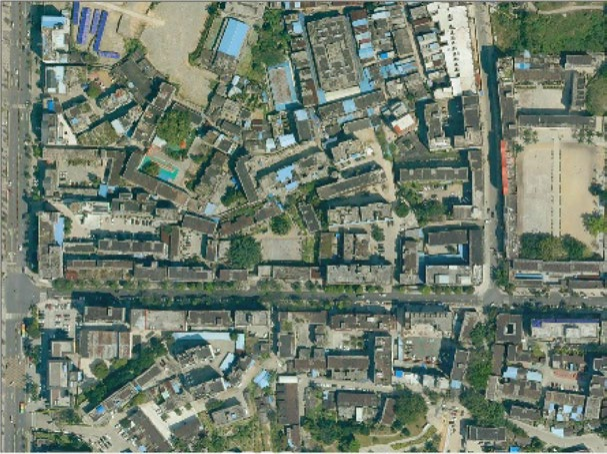

From 2007 to 2021, the average degree of habitat degradation in the central mountainous area of Hainan Island was 0.0052 and 0.0081, respectively. The degree of habitat degradation increased by 55.85%, indicating an increasing trend of habitat degradation. The dominant factor causing this trend was the intensification of agricultural reclamation and construction land development during this period, which has led to increased habitat degradation in the surrounding areas of farmland and construction land. See Figure 5.

#### 3.2.2 Changes in Habitat Quality Grades

**FIGURE 6:**
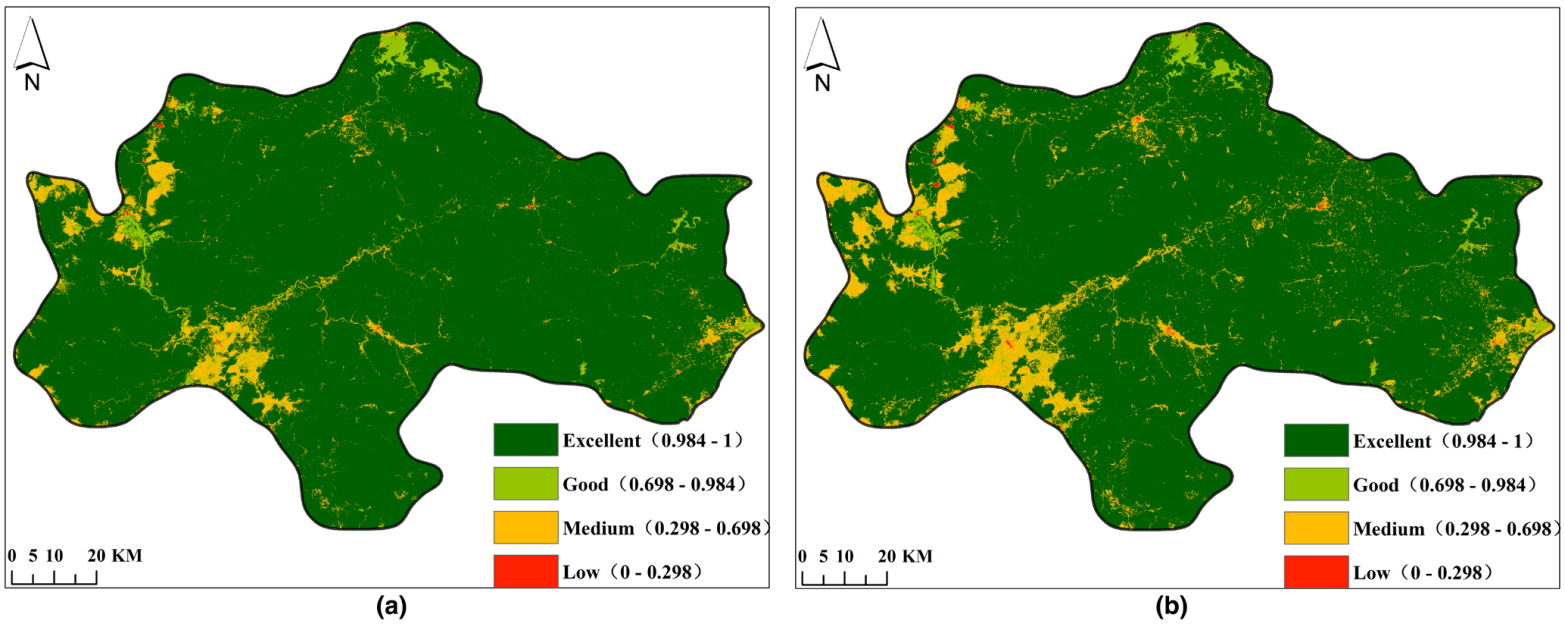
Habitat quality grade distribution map of the Central Mountainous Area of Hainan Island in 2007 (a) and 2021 (b).

In terms of habitat quality grades, from 2007 to 2021, the area of excellent habitat quality decreased from 10,377.90 km^2^ to 9924.04 km^2^, with a net decrease of 453.86 km^2^, and its proportion dropped from 92.55% to 88.51%; the area of medium habitat quality increased from 613.33 km^2^ to 1018.29 km^2^, with a net increase of 404.96 km^2^, and its proportion rose from 5.47% to 9.08%. The increment in good habitat quality area was second only to medium habitat quality, increasing from 210.37 km^2^ to 251.05 km^2^, and its proportion rose from 1.88% to 2.24%. The area of low habitat quality increased from 11.32 km^2^ to 19.52 km^2^, and its proportion rose from 0.10% to 0.17%. Over the past 15 years, the proportion of excellent habitat quality areas has always exceeded 88%, while the proportion of medium and low habitat quality areas has increased over time, indicating that the habitat quality in the entire study area was dominated by excellent grades but showed a downward trend. See Table 7, Figure 6.

**TABLE 7:** Area and proportion of habitat quality grades in the Central Mountainous Area of Hainan Island from 2007 to 2021.

Code	HQ	2007		2021		Change in 2007–2021	
		Area/km^2^	Proportion	Area/km^2^	Proportion	Area/km^2^	Proportion
4	Excellent	10,377.90	92.55%	9924.04	88.51%	−453.86	−4.37%
3	Good	210.37	1.88%	251.05	2.24%	40.69	19.34%
2	Medium	613.33	5.47%	1018.29	9.08%	404.96	66.03%
1	Low	11.32	0.10%	19.52	0.17%	8.21	72.52%

### 3.3 Scenario Simulation of Future Habitat Quality and Habitat Degradation

#### 3.3.1 LULC Simulation for 2035 Under ND and EP Scenarios

This study uses land use/land cover data from 2007 as the base period, combines 16 driving factors of land use/land cover types in the study area to simulate land use/land cover type for 2021, and repeatedly adjusts the model parameters in the PLUS model based on the results of previous studies. Comparing the prediction results for 2021 with the real land use/land cover from 2021, it was found that the Kappa coefficient, OA coefficient, and FOM coefficient were 0.68, 0.94, and 0.21, respectively, which met the simulation accuracy standards of this study. Therefore, based on the real land use/land cover type for 2021, the PLUS model was used to predict land use/land cover types for 2035.

**FIGURE 7:**
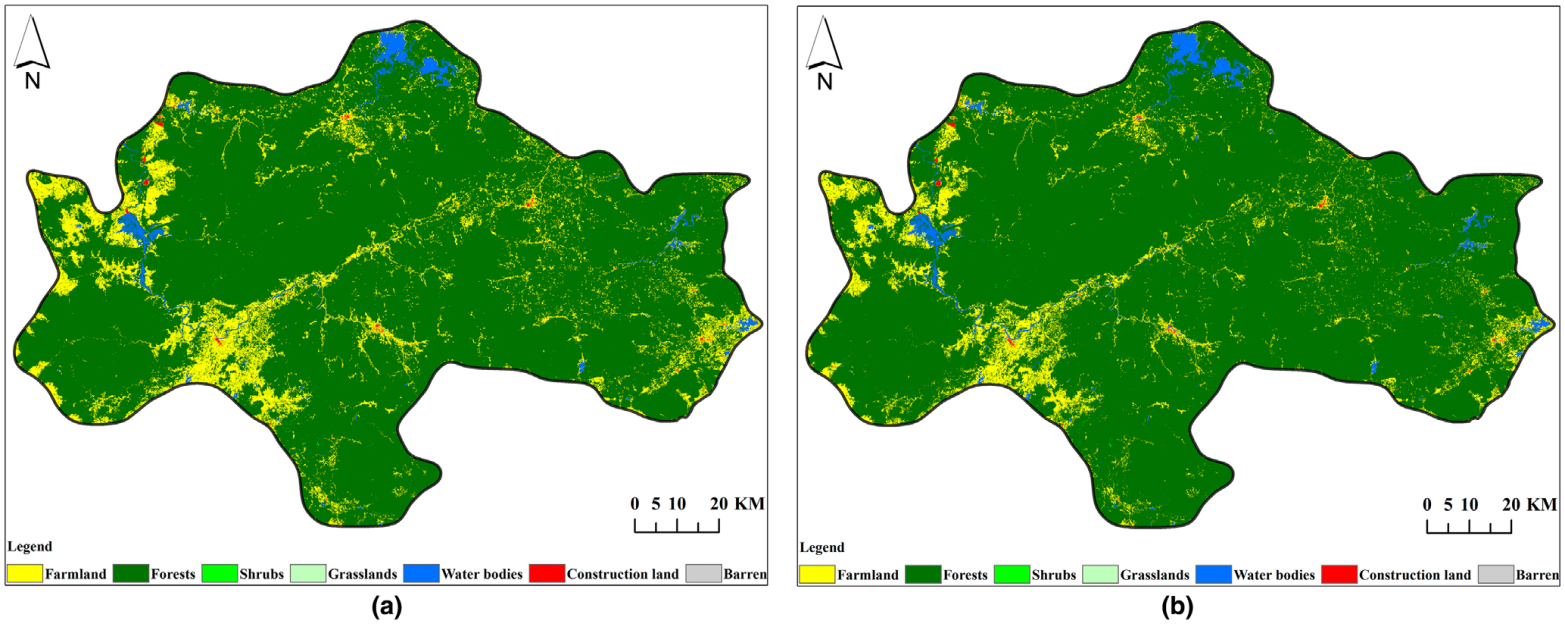
LULC (land use/land cover) distribution map of the Central Mountainous Area of Hainan Island in 2035 under ND scenario (a) and EP scenario (b).

Quantitative simulation results of land use/land cover types under ND and EP scenarios from 2021 to 2035 show that: forests and farmland are still the main land use/land cover types in the study area, which is basically consistent with the regional agricultural industrial structure and central conservation pattern. Forests dominate in both scenarios, with area ratios of 86.52% and 89.69%, respectively. During the period from 2021 to 2035, under the ND scenario, farmland will still increase significantly by 309.91 km^2^, forests will continue to decrease by 309.46 km^2^, and grasslands will be converted into water areas and farmland. Under the EP scenario, the expansion of farmland has been effectively controlled and the reduction of construction land has been promoted, with areas reduced to 62.33 km^2^ and 1.56 km^2^, respectively. The forests show an increasing trend with an increase of 45.14 km^2^. The results indicate that, in the EP scenario of the study area, the reduction of forests can be effectively controlled, while also curbing the uncontrolled expansion of farmland and promoting the reduction of construction land. See Figure 7, Table 8.

**TABLE 8:** LULC (land use/land cover) in the Central Mountainous Area of Hainan Island from 2021 to 2035 under different scenarios.

Code	LULC	2021		2035				Change in 2021–2035			
				ND scenario		EP scenario		ND scenario		EP scenario	
		Area/km^2^	Proportion	Area/km^2^	Proportion	Area/km^2^	Proportion	Area/km^2^	Proportion	Area/km^2^	Proportion
1	Farmland	1015.49	9.06%	1325.39	11.83%	953.15	8.51%	309.91	30.52%	−62.33	−6.14%
2	Forests	10,005.93	89.28%	9696.47	86.52%	10,051.07	89.69%	−309.46	−3.09%	45.14	0.45%
3	Shrubs	2.46	0.02%	2.44	0.02%	2.47	0.02%	−0.02	−0.84%	0.01	0.40%
4	Grasslands	1.72	0.02%	0.83	0.01%	1.78	0.02%	−0.89	−51.68%	0.07	4.04%
5	Water bodies	161.70	1.44%	162.23	1.45%	180.38	1.61%	0.54	0.33%	18.68	11.55%
6	Construction land	19.42	0.17%	19.34	0.17%	17.86	0.16%	−0.08	−0.39%	−1.56	−8.02%
7	Barren	0.03	0.00%	0.02	0.00%	0.01	0.00%	−0.004	−14.29%	−0.02	−67.86%

#### 3.3.2 Habitat Quality and Degradation in 2035 Under ND and EP Scenarios

**FIGURE 8:**
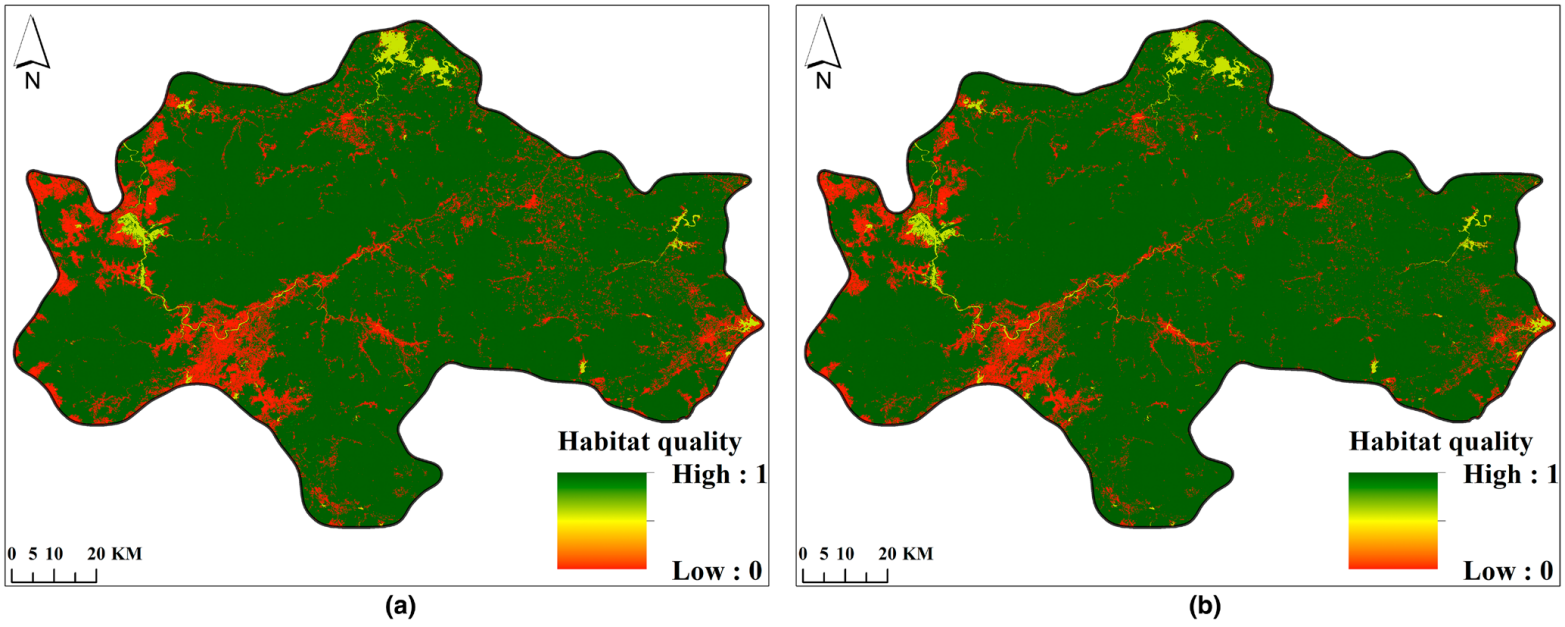
Habitat quality distribution map of the central mountainous area of Hainan Island in 2035 under ND scenario (a) and EP scenario (b).

In 2035, under the two different scenarios of ND and EP, the average habitat quality values in the central mountainous area of Hainan Island were 0.9099 (Figure 8a) and 0.9330 (Figure 8b), respectively. The habitat quality under the EP scenario was 2.53% higher than that under the ND scenario. During the period from 2021 to 2035, the habitat quality under the EP scenario showed an upward trend with a rising rate of 0.34% (Figure 4b and Figure 8b), whereas the decline rate in the ND scenario was 2.14% (Figure 4b and Figure 8a). This indicates that, compared to the ND scenario, the habitat quality is somewhat protected under the EP scenario.

**FIGURE 9:**
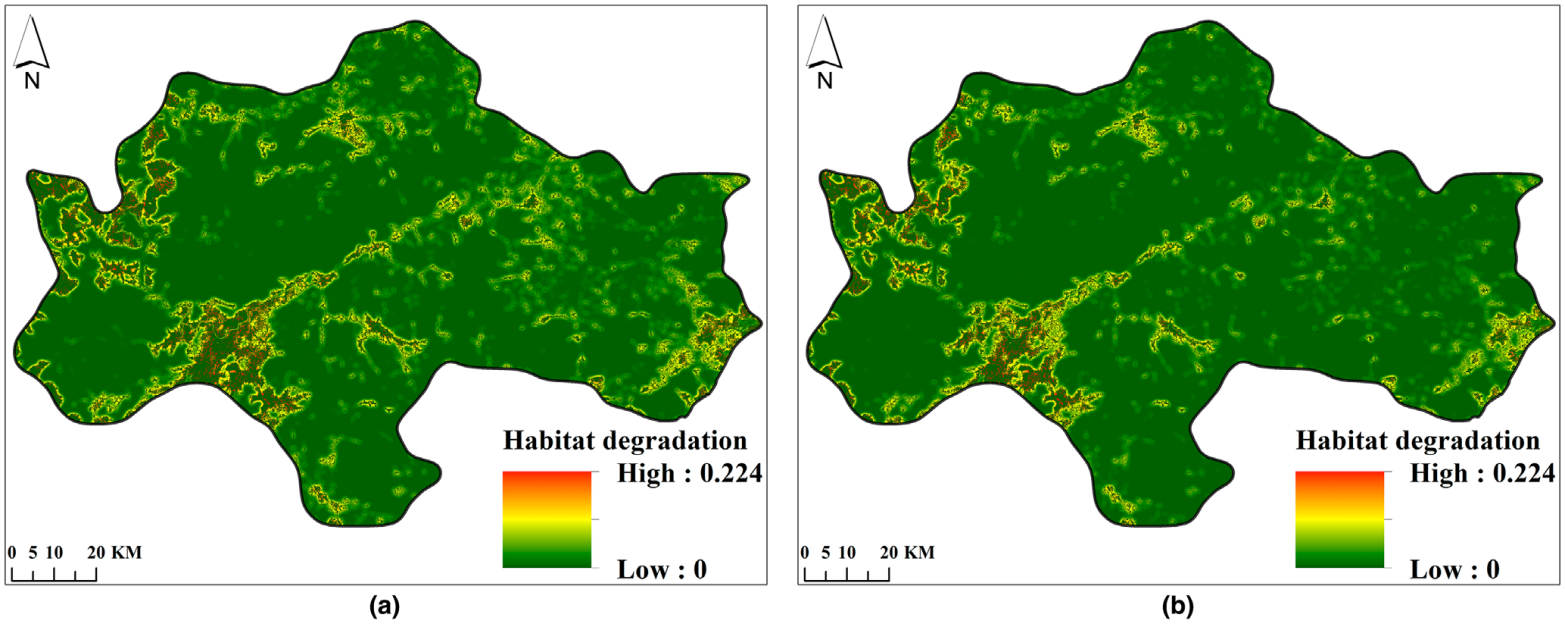
Habitat degradation distribution map of the central mountainous area of Hainan Island in 2035 under ND scenario (a) and EP scenario (b).

Under the ND and EP scenarios, the average degree of habitat degradation in the central mountainous area of Hainan Island in 2035 was 0.0100 and 0.0079, respectively. Compared to the ND scenario, the degree of habitat degradation under the EP scenario was reduced by 20.96%. The most significant decrease in habitat degradation was observed in the surrounding areas of farmland and construction land expansion. See Figure 9.

From 2021 to 2035, the rate of decline in habitat degradation under the EP scenario was 2.49% (Figure 5b and Figure 9b), whereas the increase rate under the ND scenario was 23.36% (Figure 5b and Figure 9a). This indicates that, compared to the ND scenario, the EP scenario has a certain inhibitory effect on habitat degradation.

Comparing the habitat quality grades under different scenarios in 2035, it was found that, under the ND scenario, the excellent habitat quality area was 9587.11 km^2^, accounting for 85.50% of the total area. The area of medium habitat quality was 1326.38 km^2^, accounting for 11.83% of the total area. Under the EP scenario, the excellent habitat quality area was 9981.16 km^2^, accounting for 89.01% of the total area. The area of medium habitat quality was 954.11 km^2^, accounting for 8.51% of the total area. From 2021 to 2035, compared to the ND scenario, the excellent habitat quality area of the EP scenario increased by 57.12 km^2^. The area of medium habitat quality decreased by 64.18 km^2^. The results indicate that, compared to the ND scenario, the habitat quality level in the central mountainous area of Hainan Island has improved under the EP scenario. This suggests that this scenario is more conducive to improving the habitat quality and sustainable development of the central mountainous area of Hainan Island. See Table 9.

**TABLE 9:** Habitat quality grades in the central mountainous area of Hainan Island from 2021 to 2035 under different scenarios.

Level	2021		2035				Change in 2021–2035			
			ND scenario		EP scenario		ND scenario		EP scenario	
	Area/km^2^	Proportion	Area/km^2^	Proportion	Area/km^2^	Proportion	Area/km^2^	Proportion	Area/km^2^	Proportion
Excellent	9924.04	88.51%	9587.11	85.50%	9981.16	89.01%	−336.93	−3.40%	57.12	0.58%
Good	251.05	2.24%	273.88	2.44%	253.59	2.26%	22.82	9.09%	2.54	1.01%
Medium	1018.29	9.08%	1326.38	11.83%	954.11	8.51%	308.09	30.26%	−64.18	−6.30%
Low	19.52	0.17%	25.54	0.23%	24.05	0.21%	6.02	30.83%	4.52	23.17%

## 4 Discussion

### 4.1 Driving Mechanism of LULC Changes

Changes in land use/land cover patterns in the central mountainous area of Hainan Island are the result of multiple factors working together (Jiang et al. 2013). In terms of land use/land cover evolution characteristics, the expansion of farmland and construction land is evident due to the implementation of the Hainan International Tourism Island project in 2008 and the Hainan Free Trade Port construction in 2018, which have led to rapid socio‐economic development and significantly enhanced urban construction and agricultural reclamation levels in the central mountainous area of Hainan Island. In 2022, Jin et al. pointed out that, under the influence of the launch and construction of the Hainan International Tourism Island in 2008, the overall land use changes on Hainan Island from 2008 to 2017 showed a trend of increasing farmland and construction land areas and decreasing forest land (Jin et al. 2022). A study by Xu et al. in 2024 similarly found that the area of farmland and urban ecosystems on Hainan Island showed an increasing trend from 2002 to 2020, while the area of forest ecosystems decreased (Xu et al. 2024). A study by Zheng et al. in 2019 showed that, in the ecological function protection zone of the central mountainous area of Hainan Island, the area of agricultural rubber plantations increased by 652.5 km^2^ from 1998 to 2017, while the area of natural forests decreased by 414.6 km^2^ (H. Zheng et al. 2019). Changes in land use/land cover in the central mountainous area of Hainan Island are significantly influenced by macro land development and policies. The implementation of policy factors is the dominant driver of land use/land cover structure and spatial changes (Zhou et al. 2021).

**FIGURE 10:**
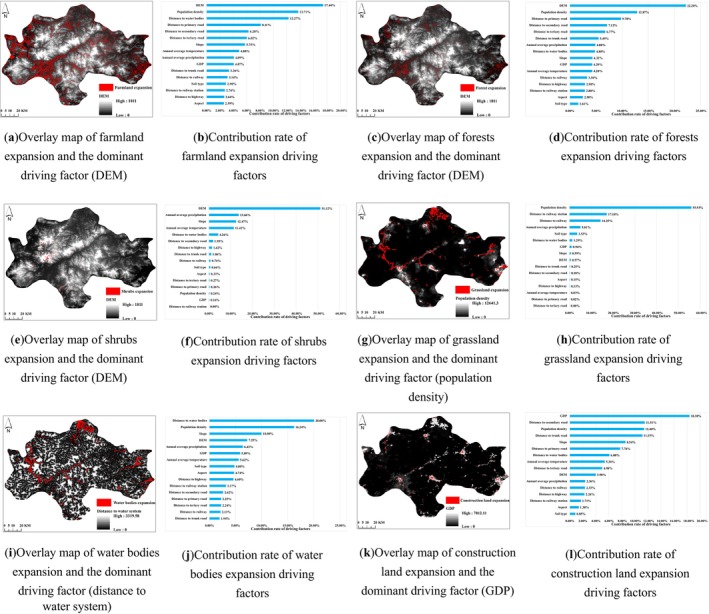
Weights of different driving factors affecting LULC (land use/land cover) change from 2007 to 2021.

Apart from the macro‐level influence of national policies, micro‐level natural factors and human activities have also contributed to changes in land use/land cover patterns (L. Chen and Wang 2021). In this study, the PLUS‐Land Expansion Analysis Strategy (LEAS) module was used to rank the driving factors of various land use/land cover changes over the past 15 years based on 16 selected factors, and a contribution analysis of land use/land cover expansion factors was conducted. This approach provides a better understanding of land use/land cover type selection and changes, offering a scientific basis for land use management and planning. After conducting an in‐depth study of the selected 16 driving factors, it was found that altitude, population density, distance to water systems, and GDP are the main factors influencing land use/land cover expansion (Figure 10). The expansion of farmland and forests is most influenced by altitude and population density, occurring in areas with lower altitudes and higher population densities. The expansion of shrubs is mainly influenced by altitude and is distributed in areas with higher altitudes. Grassland expansion is most affected by population density and mainly occurs in areas with lower population densities. The expansion area of water bodies is mainly determined by the distance to water systems and population density, occurring in areas closer to water systems and with lower population densities. The growth of construction land is mainly influenced by GDP and is concentrated in areas with higher GDP. The significant increase in farmland and construction land indicates a notable enhancement in human land reclamation and development utilization. The expansion of farmland and construction land poses a significant threat to the habitat quality of the central mountainous areas of Hainan Island.

### 4.2 The Impact of LULC Changes on Habitat Quality

The land use/land cover system is a complex ecosystem, and human activities have an important impact on its development and changes. Meanwhile, the comprehensive impact of this system also affects humans (Li, Dong, et al. 2022). The expansion of farmland and construction land in the study area from 2007 to 2021 is closely related to the socio‐economic situation. Among all land use/land cover types, farmland and construction land have a more pronounced impact on habitat quality. Threat factors to habitat quality include farmland and construction land. Changes in the area of these two land use/land cover types directly affect changes in habitat quality (Xu et al. 2023). Therefore, we can predict that, in key biodiversity areas, a balance can be achieved between socio‐economic development and habitat quality protection. Combining the actual situation of the central mountainous area of Hainan Island, this study area evaluates the restricted areas of forest, shrubland, grassland, and water area conversion within the Ecological Conservation Redline using the PLUS‐InVEST model, in accordance with strict regulatory requirements for the Ecological Conservation Redline. The results of this study show that the expansion of farmland and construction land leads to a decline in regional habitat quality, which once again verifies the research results of Wei et al. (2022) and Tang et al. (2020). However, the area of farmland is still expanding. How to optimize land resources and restore ecological protection under the current situation in the central mountainous area of Hainan Island is an issue that needs attention.

### 4.3 Advantages of the Model in the Study

The InVEST model adopted in this study is widely used due to its fewer parameter settings and stronger visualization capabilities (Wang and Cheng 2022). In addition, the model is relatively mature and has certain advantages over other traditional methods in terms of spatial expression and dynamic analysis research (Wu et al. 2024). In this study, the remote sensing data‐driven PLUS‐InVEST model integrates a new Land Expansion Analysis Strategy (LEAS) and a Cellular Automata (CA) model based on a new multiple random seeds mechanism, enabling high‐precision simulation of patch changes for different types of land use/land cover. Compared to other models such as FLUS, the PLUS model has higher accuracy in simulating land use/land cover type changes, effectively addressing the issue of simulation data accuracy in large‐scale study areas (Z. Lin and Peng 2022; Wang, Zhang, et al. 2022).

### 4.4 Limitation in the Study

In this study, the dynamics of land use/land cover, habitat quality, and habitat degradation in the central mountainous area of Hainan Island from 2007 to 2021 were explored. The remote sensing data‐driven PLUS‐InVEST model was used to predict the changes in land use/land cover, habitat quality, and habitat degradation under two scenarios in 2035. It provides a reference for improving habitat quality and reducing habitat degradation in the central mountainous area of Hainan Island. However, this study has some limitations. Only land use/land cover type data, population density, GDP, transportation facilities, temperature, precipitation, DEM, slope, aspect, soil type, and water systems were considered. Other factors that affect socio‐economic construction, climate and environment, and human activities were not fully considered. Therefore, it is necessary to further enrich and improve the indicator system.

## 5 Conclusions

The central mountainous area of Hainan Island, as one of the key areas of global biodiversity, has particularly important ecological functions. In terms of habitat quality, any increase or decrease in each land use/land cover type in the central mountainous region of Hainan Island will affect its changes. With the increasing impact of human activities, significant changes have occurred in the habitat quality of the central mountainous area of Hainan Island. The results show that, from 2007 to 2021, farmland and construction land increased, while forests, shrubs, and grasslands decreased, resulting in significant changes in the spatial distribution pattern of habitat quality. Farmland and construction land are the main factors affecting habitat quality in the study area, and their increase or decrease affects the improvement and decline of habitat quality in the study area. Combining the scope of the Ecological Conservation Redline, a comparative analysis of land use/land cover distribution patterns and changes in habitat quality under different scenarios in 2035 was conducted. The research results indicate that, under the ecological priority (EP) scenario, the expansion of farmland and the shrinkage of forests have been effectively curbed, while the reduction of construction land has been promoted, leading to an improvement in habitat quality.

Although this study constructed a remote sensing‐driven PLUS‐InVEST model that analyzed the impact mechanisms of land use/land cover change on habitat quality, the indicator system emphasized natural geographic factors (e.g., elevation, soil type) and basic socioeconomic data (e.g., population density, GDP), yet inadequately considering climate change‐derived factors (e.g., extreme climate event frequency, spatiotemporal heterogeneity of CO_2_ concentration) and micro‐mechanisms of human activities (e.g., land policy implementation efficiency, community ecological compensation effects), etc.

To address these limitations, we recommend establishing an extended indicator system incorporating climate variability factors, policy intervention variables, and community participation mechanisms, etc., combined with structural equation modeling (SEM), geographical detector (GeoDetector), and other methods to analyze interactions among multidimensional driving factors. Additionally, based on the above main findings, it is recommended to conduct more in‐depth research on the optimization of land use/land cover structure in the central mountainous area of Hainan Island, aiming to provide a more universal scientific paradigm reference for ecosystem conservation, habitat quality improvement, and sustainable development in similar island tropical rainforest areas.

## Author Contributions

**Jiceng Xu:** conceptualization (lead), data curation (lead), formal analysis (lead), investigation (lead), methodology (lead), software (lead), validation (lead), visualization (lead), writing – original draft (lead). **Xiaodong Mu:** resources (supporting), supervision (lead), writing – review and editing (lead). **Ziwei Ma:** software (supporting).

## Conflicts of Interest

The authors declare no conflicts of interest.

## Data Availability

Data from the results of this study are freely available at https://doi.org/10.6084/m9.figshare.28529279.
